# Implementing the teen marijuana check-up in schools—a study protocol

**DOI:** 10.1186/s13012-017-0633-5

**Published:** 2017-08-10

**Authors:** Bryan Hartzler, Aaron R. Lyon, Denise D. Walker, Lauren Matthews, Kevin M. King, Kathryn E. McCollister

**Affiliations:** 1 Alcohol and Drug Abuse Institute, University of Washington, 1107 NE 45th Street, Suite 120, Seattle, WA 98105 USA; 2 Psychiatry and Behavioral Sciences, University of Washington, 6200 NE 74th Street, Suite 100, Seattle, WA 98105 USA; 3 School of Social Work, University of Washington, 909 NE 43rd Street, Suite 304, Seattle, WA 98105 USA; 4 Department of Psychology, University of Washington, 119A Guthrie Hall, Seattle, WA 98195 USA; 5 Department of Public Health Sciences, University of Miami Miller School of Medicine, 1120 NW 14th Street, Suite 1019, Miami, FL 33136 USA

**Keywords:** Technical assistance, Therapy training, EBP implementation, Fidelity, Motivational enhancement therapy, Adolescent marijuana use

## Abstract

**Background:**

Substance misuse is now encountered in settings beyond addiction specialty care, with schools a point-of-contact for student access to behavioral health services. Marijuana is a leading impetus for adolescent treatment admissions despite declining risk perception, for which the Teen Marijuana Check-Up (TMCU)—a tailored adaptation of motivational enhancement therapy—offers an efficacious service option. To bridge the knowledge gap concerning effective and affordable technical assistance strategies for implementing empirically supported services, the described trial will test such a strategy to facilitate school-based TMCU implementation.

**Methods:**

A type II effectiveness/implementation hybrid trial will test a novel strategy for a TMCU purveyor to provide technical assistance on an ‘as-needed’ basis when triggered by a fidelity drift alarm bell, compared to resource-intensive ‘gold-standard’ technical assistance procedures of prior efficacy trials. Trial procedures adhere to the EPIS framework as follows: (1) initial mixed-method *exploration* of the involved school contexts and identification of TMCU interventionist candidates in elicitation interviews; (2) interventionist *preparation* via a formally evaluated training process involving a two-day workshop and sequence of three training cases; (3) post-training *implementation* for 24 months for which trained interventionists are randomized to ‘as-needed’ or ‘gold-standard’ technical assistance and self-referring students randomized (in 2:1 ratio) to TMCU or waitlist/control; and (4) examination of TMCU *sustainment* via interventionist completion of biannual outcome assessments, cost analyses, and exit interviews. Hypothesized effects include non-differential influence of the competing technical assistance methods on both TMCU fidelity and intervention effectiveness, with lesser school costs for the ‘as-needed’ than ‘gold-standard’ technical assistance and greater reduction in the frequency of marijuana use expected among TMCU-exposed students relative to those assigned to waitlist/control.

**Discussion:**

This trial—occurring in Washington state as legislative, fiscal, and sociocultural forces converge to heighten exposure of American adolescents to marijuana-related harms—is set to advance understanding of best implementation practices for this and other efficacious, school-based interventions through examination of a data-driven technical assistance method. If shown to be clinically useful and affordable, the concept of a fidelity drift alarm could be readily translated to other empirically supported services and in other health settings.

**Trial registration:**

ClinicalTrials.gov NCT03111667 registered 7 April 2017.

## Background

Traditional health service research processes contribute to a 17-year lag between empirical validation and community implementation [1–3]. A common caveat of trials that establish efficacy of a given health service is the resource-intensive manner in which selected research therapists are trained and supervised to ensure the health service is delivered with fidelity so its hypothesized effects are adequately demonstrated [4]. Replication of these training and technical assistance procedures to support community-based implementation is unrealistic in most settings. Consequent need for effective, affordable strategies to support community implementation of empirically supported health services casts a shadow over the prospect of their transport to community settings, including those wherein substance misuse is encountered. Notably, legislative policies in the United States of America (i.e., Mental Health Parity and Addiction Equity Act, 2008; Patient Protection and Affordable Care Act, 2010) have diversified such settings [5].

### ‘Science-to-practice gaps’ in the addiction field extend to adolescent treatments and settings

Dating back two decades to a widely cited Institute of Medicine report [6], a persisting focus in the addiction field is bridging ‘science-to-practice gaps.’ As estimated by the 2014 National Survey on Drug Use and Health, a majority of the 1.3 million American adolescents with a substance use disorder struggle with marijuana specifically [7]. Further, marijuana is a leading impetus for treatment admissions [8], and linked to lesser neuropsychological functioning and academic performance [9] and greater likelihood of school dropout, emergent psychopathology, and suicidality [10, 11]. Nevertheless, risk perceptions about marijuana use have declined among high school students [12], perhaps a function of increasingly progressive legislation widening its public availability and permissibility [13]. Consequent demand for cost-effective, clinically useful marijuana-focused treatments for adolescents is high.

Historically, addiction research has focused on adults, as evident in prominent trials comparing empirically supported therapies [14] or evaluating their combination with medications [15]. Also of primary focus has been the addiction specialty care context, as evidenced in three dozen multisite protocols conducted by the National Institute on Drug Abuse via its Clinical Trials Network [16]. Expanded inclusion of substance use disorders among essential conditions for which Americans may access health services has prompted treatment-seeking in other settings that serve as a point-of-contact [17]. Schools are an example as 70–80% of adolescents who access services doing so in schools [18]. Most school-based services lack empirical support [19, 20], and introduction of empirically supported services (if effectively implemented by school personnel) may substantively improve public health efforts [21, 22].

### Conceptual and empirical basis for the teen marijuana check-up

Among prominent evidence-based therapies is Motivational Interviewing (MI), a “collaborative conversation style for strengthening a person’s own motivation and commitment to change” [23]. An oft-studied brief adaptation of MI is Motivational Enhancement Therapy (MET), typically structured as 1–2 individual sessions wherein personal assessment data is gathered and explored [24]. This brief structure and hallmark client-centered care philosophy were core features of a popular alcohol-focused approach, the Drinker’s Check-Up [25], which spurred similar self-referral interventions for specific populations and behaviors [26]. Among these is a school-based intervention targeting marijuana use [27], the Teen Marijuana Check-Up (TMCU), since empirically validated [28–30] and designated as an evidence-based practice [31]. Table 1 lists its core characteristics.

**Table 1:** Intervention characteristics of the teen marijuana check-Up

Intervention characteristic	
Therapeutic foundation	Motivational interviewing
Target population	Teens, aged 14–19 years
Nature of recruitment	Self-referral
Implementation setting	High schools
Intervention format	Brief individual therapy
Intervention duration	Two 60-min sessions, scheduled approximately 1 week apart
Core features	Patient-centered communication, review of a personalized feedback report
Primary outcomes	Decreased marijuana use, reduced marijuana-related harms
Secondary outcomes	Improved academic functioning, increased school engagement

TMCU characteristics as developed in or informed by a series of prior efficacy trials [31–34]

### A key challenge for implementing TMCU in schools

As in other health fields, experimental controls inherent in adolescent treatment trials limit generalizability of resulting findings [32]. This is prominent among reasons for ‘voltage drop,’ where effect size diminishes upon a therapy’s community implementation [33]. Though public demand for school-based services like TMCU is strong, the prospect of real-world TMCU implementation raises concerns about identification, training, and oversight of school-based personnel. Extant research on MI training conducted with a diverse workforce documents: (1) insufficiency of workshop training to cull durable skills [34, 35], and (2) utility of post-workshop technical assistance via coaching and performance-based feedback [36–38]. While such technical assistance is broadly recommended to disseminate health services [39–41], key questions for schools at which TMCU is implemented are how much technical assistance personnel require.

Given prominent concern about costs [42, 43], much may be gained from identifying affordable strategies to avail technical assistance [44]. In efficacy trials, gold-standard technical assistance includes fidelity monitoring, provision of feedback on work-samples, and recurrent behavioral rehearsal opportunities [45]—all to assure research therapists maintain sufficient fidelity [46]. Such gold-standard technical assistance was included for research therapists in TMCU efficacy trials [27–30]. Given scant resources at many schools, the prospect of replicating such procedures in support of TMCU implementation is understandably daunting. A consequent challenge is to identify alternative strategies whereby technical assistance is offered to resource-challenged schools without compromise to TMCU fidelity [47].

### Process control benchmarking: a promising TMCU implementation support strategy

One potential option for affordable, effective technical assistance in implementing the TMCU is governance by process control benchmarking. This involves continual tracking of interventionist fidelity and comparison to a priori benchmarks [48], whether conceptually derived performance standards [49], norms derived from a clinical database [50], or aggregated efficacy trial data [51]. For the TMCU, this comparative process will be facilitated by existence of conceptually derived performance standards for MI/MET interventions [52] and fidelity data from efficacy trials encompassing a dozen research therapists and nearly 700 students [27–30].

**Fig. 1:**
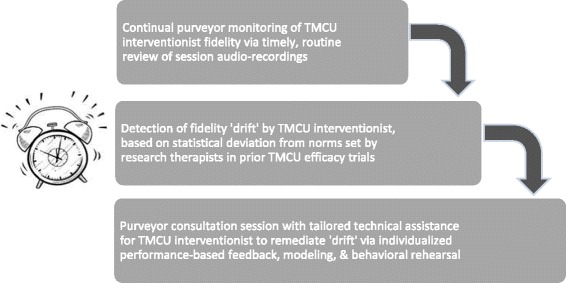
Fidelity drift alarm

A testable implementation support strategy utilizing process control benchmarking, aforementioned MI/MET performance standards, and prior TMCU efficacy trial data is a *fidelity drift alarm* [48], illustrated in Fig. 1. As school-based interventionists are subject to natural performance variance due to setting demands, student case-mix, and situational factors, a TMCU fidelity drift alarm would signal points of deviation from acceptable fidelity parameters, with an alarm bell prompting individually focused technical assistance. If occurring situationally, or ‘as-needed,’ this is likely to diminish required resources. A fidelity drift alarm may be maintained (and re-calibrated, if appropriate) over time, thus allowing malleable and continual fidelity-monitoring. Relative to gold-standard technical assistance, this may be viable as a clinical and cost-effective method of technical assistance for schools to transition to lesser purveyor reliance. Moreover, it represents a sustainment strategy that may be applied to other empirically supported therapies implemented in schools.

The trial design described herein reflects a protocol funded by the National Institute on Drug Abuse (R01 DA040650, A Hybrid Effectiveness-Implementation Trial of a School-Based Teen Marijuana Check-Up). A hybrid type II trial [53], co-primary aims are to compare technical assistance methods for school-based TMCU interventionists on implementation outcomes and intervention effectiveness. School leaders will identify staff to be trained and serve as interventionists, then randomized to receive technical assistance over a 2-year period via (1) a ‘gold-standard’ group session occurring weekly, or (2) ‘as-needed’ individual sessions, triggered by a fidelity drift alarm calibrated by process control benchmarking. Self-referring students will be randomized, in 2:1 ratio, to TMCU or waitlist/control. Hypothesized effects are for non-differential impact of technical assistance methods on interventionists’ TMCU fidelity and marijuana use among TMCU-exposed students. Other expectations are for lesser cost of ‘as-needed’ technical assistance, and clinical effectiveness of TMCU relative to waitlist/control.

## Methods/design

This hybrid type II effectiveness/implementation trial models the ‘Exploration, Preparation, Implementation, Sustainment’ (EPIS) framework [54]. This includes: mixed-method exploration of school contexts, preparation of school-based staff via formally evaluated TMCU training, post-training implementation over 24 months wherein trained TMCU interventionists are randomized to receive one of two technical assistance methods, and examination of sustainment via interventionist completion of biannual outcome assessments. As legislative and sociocultural factors converge in Washington state that heighten adolescent exposure to potential marijuana-related harms, this trial seeks to advance knowledge of best implementation practices for this and other school-based interventions.

### Units of analysis and measures

Trial measurement includes students nested in interventionist caseloads, which in turn are nested in schools. Units of analysis exist at three levels: school, interventionist, and student. Corresponding data collection instruments are listed in Table 2.

**Table 2:** Trial measurement instruments and processes

Unit of analysis	Instrument/process	Respondent(s)	Timing of assessment
Schools			
	Implementation leadership scale [62]	School staff members	Trial outset
	Implementation climate scale [63]	School staff members	Trial outset
	Site visit including elicitation interview with school leadership	Principal, other leaders	Trial outset
	Exit interview	Principal, other Leaders	Trial conclusion
Interventionists			
	Demography and background	Interventionists	Pre-training
	MI knowledge/attitudes test [64]	Interventionists	Pre/post-training + 6-, 12-, 18-, 24-month
	Adoption readiness scale [65]	Interventionists	Pre/post-training + 6-, 12-, 18-, 24-month
	Fidelity-rated interaction with a standardized patient [66]	Interventionists	Pre/post-training + 6-, 12-, 18-, 24-month
	Training satisfaction survey	Interventionists	Post-training
	TA satisfaction survey	Interventionists	6-, 12-, 18-, 24-month
	Interventionist time-log	Interventionists	Weekly, over 24 months
Students			
	Demography and locator data	Students	Baseline
	Normative perceptions [67, 68]	Students	Baseline
	Life goals [69]	Students	Baseline
	Global Appraisal of Independent Needs [70]	Students	Baseline, 3- and 6-month
	Marijuana Problems Scale [71]	Students	Baseline, 3- and 6-month
	Marijuana Motives Measure [72]	Students	Baseline, 3- and 6-month
	Self-efficacy to Avoid Marijuana [73]	Students	Baseline, 3- and 6-month
	Student Academic Self-Report [74]	Students	Baseline, 3- and 6-month
	Student Engagement Instrument [75]	Students	Baseline, 3- and 6-month
Investigators			
	Technical Assistance Time-Log	Purveyor	Weekly, over 24 months
	Stages of Implementation Completion Checklist [76]	Research coordinator	Continual

Trial design proposes recruitment of approximately ten schools, with 2–4 interventionists recruited per participating school to garner an intended sample of TMCU interventionists (*N* = 30) randomized to ‘gold-standard’ or ‘as-needed’ technical assistance conditions; Self-referring students (*N* = 250) will be randomized in a 2:1 ratio to receive the TMCU intervention vs. waitlist/control

#### Schools

Trial measurement at a school-level is governed by a mixed-method approach, predominantly initially assessment of school attributes. For an estimated ten participating high schools, this includes web-based surveys of strategic leadership [55] and implementation climate [56] completed by school staff (not restricted to those serving as TMCU interventionists). This is supplemented by school data necessary for determination of TMCU costs (i.e., existing resources for substance use programming; interventionists’ salary; required facilities, equipment, supplies). This data will be gathered qualitatively in an initial elicitation interview with school leadership. Similar interviews are conducted at trial conclusion with school leadership and other personnel, focused on perceived facilitators and barriers for TMCU sustainment.

#### Interventionists

Trial measurement for 30 TMCU interventionists to be recruited (*n* = 2–4 per school) includes pre-training collection of demography (age, gender, ethnicity, race), professional background (educational attainment; setting tenure, role, duties), and prior MI exposure. Repeated-measures assessed in six training outcome assessments (i.e., pre/post-training; 6-, 12-, 18-, 24-month follow-up) are the survey-based Motivational Interviewing Knowledge and Attitudes Test [57] and Adoption Readiness Scale [58], which supplement an audio-recorded standardized patient (SP) interaction conducted in interventionists’ workspace and independently scored with a validated fidelity scale [59] by raters blinded to technical assistance condition. Post-training assessment specifically adds a web-based survey of training satisfaction, with follow-up assessments similarly tapping satisfaction with technical assistance and potential contamination between technical assistance methods. To aid planned cost analyses, interventionists will submit weekly time-logs to document effort in core implementation activities (i.e., hours spent recruiting students and delivering the intervention). Interventionists will receive financial remuneration to incentivize retention and completion of assessments.

#### Students

Trial measurement of 250 high school students to self-refer for randomization (in 2:1 ratio) to TMCU or waitlist/control includes baseline survey of demography, contact information, normative perceptions of marijuana use [60, 61], and life goals information [62] incorporated as personal feedback. Repeated-measures assessed in intervention outcome assessments (i.e., baseline, 3- and 6-month follow-up) include the following student-report instruments (1) Global Appraisal of Independent Needs [63], offering recent marijuana use and diagnostic symptoms; (2) Marijuana Problems Scale [64]; (3) Marijuana Motives Measure [65]; (4) self-efficacy to avoid marijuana use [66]; (5) Student Academic Self-Report, tapping homework completion, attendance, and disciplinary actions [67]; and (6) Student Engagement Instrument [68], measuring academic and social engagement. This will be supplemented by administrative data accessed from participating school districts. Students will receive financial remuneration to incentivize retention and completion of outcome assessments.

#### Other measurement

Trial measurement includes two staff-report instruments. To facilitate cost analyses, the TMCU purveyor (DW) will over the 24-month implementation period complete a weekly time-log of technical assistance efforts. Also, a Stages of Implementation Completion checklist [69], will be continually updated by the trial coordinator (LM) with record of milestone dates at participating schools for engagement, feasibility determination, trial planning, interventionist training, introduction of technical assistance, initiation of TMCU services, fidelity-monitoring, school decisions about sustainment.

### Trial procedures

A procedural chronology begins with initial exploration of participating school contexts. This involves recruitment of schools, assessment of their setting attributes, and identification of staff candidates to serve as TMCU interventionists.

#### School recruitment

An initial step to access the intended sample of 30 interventionists is recruitment of schools, a process governed by the TMCU purveyor (DW) with whom local schools previously collaborated. Initially, school district-level approval will be sought, followed by contacts to school principals. Although no formal inclusion/exclusion criteria constrain this process, common contextual conditions are expected (i.e., need for marijuana-focused student services, interested and capable staff to serve as interventionists). The number of schools recruited is guided by access to a sufficient number of interventionists (*N* = 30) for planned comparison of ‘gold-standard’ and ‘as-needed’ technical assistance. Consideration is also given to schools’ census, so a requisite number of self-referring students (*N* = 250) is achieved.

#### Assessment of school attributes

Two investigative team members (AL, DW) will schedule a site visit to observe setting attributes germane to TMCU implementation costs and conduct an audio-recorded elicitation interview with school leadership. The purpose of the interview is to (1) confirm understanding of trial aims and procedures; (2) gather information concerning school mission and resources, organizational climate and culture, structure and size of existing staff, and expectations and concerns about TMCU implementation; and (3) elicit a shortlist of TMCU interventionist candidates and their salary information. School leaders will review and sign a consent form prior to the interview. Interview recordings will be coded by the investigative team using conventional and directed content analysis [70]. Interested school staff will review and sign a consent form and complete a web-based survey tapping perceptions of strategic leadership [55] and implementation climate [56].

#### Identification of TMCU interventionists

**Fig. 2:**
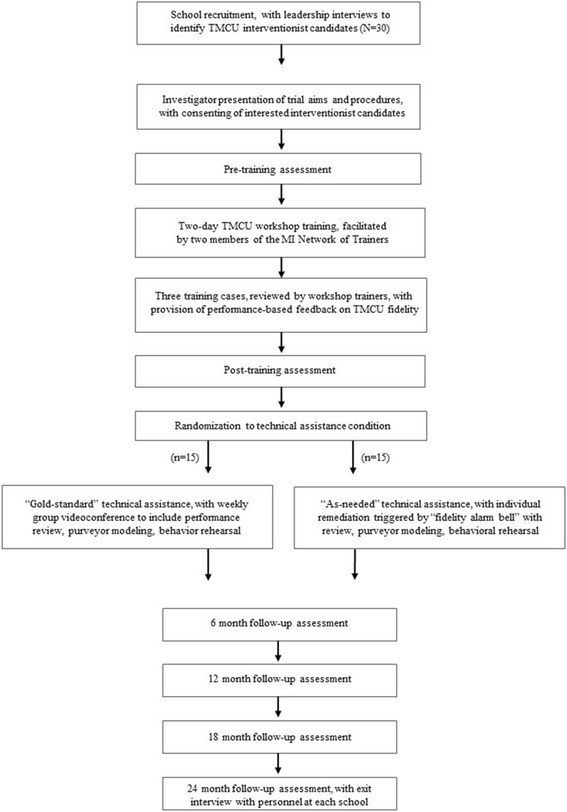
CONSORT flow diagram for TMCU interventionists

Identified TMCU interventionist candidates will attend an on-site presentation. In addition to orientation to TMCU, the presentation will outline trial benefits (i.e., purveyor-led training and technical assistance, continuing education units) and expectations (i.e., completion of serial training outcome assessments, school designation as a TMCU interventionist, on-site promotion, screening and intervention, session audio-recording, submission of time-logs). Interested staff will be invited to become TMCU interventionists and asked to review and sign a corresponding consent form. Figure 2 offers a CONSORT flow diagram depicting trial procedures for TMCU interventionists.

The second EPIS phase involves preparation for school-based staff to serve as TMCU interventionists, for whom broad inclusion criteria should increase generalizability of trial findings. This includes their completion of a pre-training assessment, workshop training plus training cases, and a post-training assessment. Trained staff will then be randomized to one of two technical assistance methods.

#### Pre-training assessment

Interventionists will complete a web-based survey assessment of personal demography and professional background, MI knowledge and attitudes, and adoption readiness. Interventionists will also complete an audio-recorded encounter with a standardized patient (SP), who remains in character during portrayal of a marijuana-using teen. As in all training outcome assessments, the trial coordinator (LM) will coordinate scheduled times for SP encounters, with resulting audio-recordings independently scored for fidelity.

#### Workshop training

Identified staff will complete workshop training with a curriculum to mirror that used in TMCU efficacy trials. Readings will be provided in advance, to include research on marijuana and its effects, the TMCU manual, and an MI text [71]. Two investigators (BH, DW), both longstanding Motivational Interviewing Network of Trainers (MINT [72]) members, will facilitate a 2-day workshop with didactic review of MI principles and the TMCU manual, modeling of techniques, and behavioral rehearsal exercises. The initial workshop day is devoted to research findings on marijuana, risk factors for substance abuse, and introduction to MI principles. The latter workshop day focuses on the TMCU approach, with detailed review of the TMCU personal feedback report. Relevant MI skills will be introduced and practiced, with sample case vignettes offering further opportunities for TMCU interventionists to practice skills.

#### Standardized training cases

Following the workshop, each TMCU interventionist will complete training cases involving SP portrayal of a marijuana-using teen. Each case will be structured as a 30–45 min interaction, to occur at arranged times in interventionists’ regular workspace. The workshop trainers (BH, DW) will score corresponding audio-recordings with an MI fidelity instrument [73], providing written feedback on performances relative to these conceptually derived behavioral fidelity benchmarks (1) reflection-to-question ratio (R:Q), (2) percent open questions (%OQ), and (3) percent complex reflections (%CR).

#### Post-training assessment

Upon training conclusion, interventionists complete a web-based survey assessment of MI knowledge and attitudes, adoption readiness, and training satisfaction. Additionally, interventionists will repeat the audio-recorded SP encounter to assess the impact of training on TMCU fidelity.

#### Staff randomization

At training conclusion, the trial coordinator (LM) will allocate via urn randomization TMCU interventionists to receive technical assistance via (1) a ‘gold-standard’ weekly group session, or (2) ‘as-needed’ individual sessions triggered by fidelity drift alarm. A shared feature of both methods is the direct oversight of the TMCU purveyor (DW), who will monitor fidelity in review of session audio-recordings and provide numeric feedback to interventionists regarding behavioral fidelity indices (i.e., R:Q, %OQ, %CR). Notably, the technical assistance methods differ in format and anticipated frequency of occurrence, with differential cost expected.

**Fig. 3:**
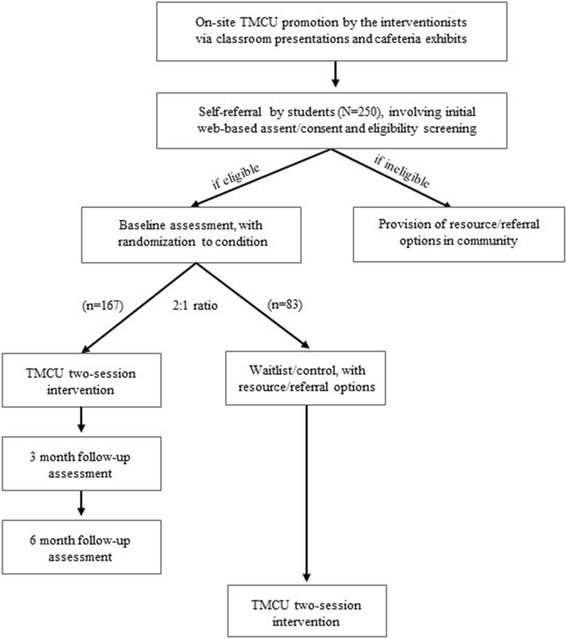
CONSORT flow diagram for students

The third EPIS phase focuses on TMCU implementation at participating schools over a 24-month period. This includes recruitment and randomization of self-referring students, baseline student assessment, TMCU delivery, fidelity-monitoring and technical assistance, and student outcome assessments (3-, 6-month follow-ups). To inform cost-benefit and cost-effectiveness analyses, both purveyor and interventionists complete weekly continual time-logs documenting effort in select activities. Figure 3 offers a CONSORT flow diagram depicting trial procedures for self-referring students.

#### Student recruitment

At each school, TMCU interventionists will contribute to on-site promotion via classroom presentations and cafeteria exhibits. Self-referring students will have a TMCU interventionist read a brief explanation of the trial, and if interested will be directed to a web-based assent/consent form with contact information for university-based research staff. Students will provide informed assent/consent prior to interventionist conduct of a brief, confidential screening interview to determine eligibility. Inclusion criteria are that students be (1) 14–19 years old, (2) enrolled in 9th–12th grade, and (3) active marijuana users (9+ days in prior month). Students will be excluded if they are not fluent in English, have a thought disorder, or refuse to be randomized to condition. Ineligible students will receive standard marijuana resources/referrals.

#### Student randomization

Upon determination of eligibility, students will be randomized in a 2:1 ratio to TMCU or waitlist/control. This will occur after blocking by student gender and grade-level, independently at each school. The trial seeks 250 student participants during its 24-month period of TMCU implementation.

#### Baseline student assessment

All student assessments occur over encrypted web servers. Baseline assessment will gather demography, contact information, and TMCU personal feedback report data on marijuana use, problems, motives, perceptions, and self-efficacy to abstain.

#### TMCU delivery

Trained interventionists will deliver the TMCU to eligible, self-referring students over a 24-month period. This consists of two sessions, each 45 min in duration occurring a week apart. The initial session develops rapport, explores aspects of the student’s marijuana use, and identifies sources of motivation to change. The latter session includes conjoint review of the personal feedback report, with the interventionist eliciting self-motivational statements about reduction or discontinuance of marijuana use and offering advice for such changes. Notably, interventionists deliver TMCU to waitlist-assigned students only after a requisite 6-month intervening period.

#### Purveyor fidelity-monitoring

All TMCU sessions will be subject to fidelity-monitoring by the purveyor (DW). Fidelity will be evaluated via a behavioral coding system with demonstrated validity for evaluating adherence of MI-based interventions [73]. It includes continuous counts of specific interventionist behaviors from which three focal behavioral fidelity indices are computed (i.e., R:Q, %OQ, %CR).

#### ‘Gold-standard’ technical assistance

Modeled after efficacy trial procedures, TMCU interventionists assigned to this condition will attend a weekly videoconference. The purveyor (DW) will lead discussion of her review of behavioral fidelity indices and excerpts of recently conducted sessions. If interventionist drift is identified in a reviewed session, this will prompt (1) conceptual review of relevant TMCU skills, (2) purveyor demonstration, and (3) behavioral rehearsal by the interventionist.

#### ‘As-needed’ technical assistance

The purveyor (DW) will initiate individual sessions by videoconference at points when the fidelity alarm bell is triggered by unacceptable drift in one or more behavioral fidelity indices (i.e., R:Q, %OQ, %CR). The alarm will be calibrated using process control benchmarking, with interventionist performances compared against index distributions of research therapists from efficacy trials. For each fidelity index, these distributions will specify an a priori lower control limit to reflect the poorest acceptable TMCU fidelity. Interventionist performances that breach such limits will trigger the alarm, prompting ‘as-needed’ remediation. Technical assistance activities will mirror those of ‘gold-standard’ sessions (i.e., conceptual review, purveyor demonstration, interventionist behavioral rehearsal).

#### Student outcome assessments

The trial coordinator will govern procedures for these assessments at 3- and 6-month follow-up points, for which the purpose is to evaluate TMCU’s clinical effectiveness. Students will similarly complete follow-up web-based assessments on encrypted internet server. Attrition will be minimized through use of extensive contact/locator information by research staff, who maintain networking relationships with local social service agencies. Similar procedures have led to 90+% retention rates in prior TMCU trials.

#### Purveyor/interventionists time-logs

To inform eventual cost analyses, the purveyor and interventionists submit weekly time-logs during the 24-month implementation period. The purveyor documents effort in fidelity-monitoring, individual/group technical assistance, and crisis consultation or other interventionist support activities. The TMCU interventionists document effort recruiting students, delivering TMCU sessions, and attending technical assistance sessions.

The final EPIS phase addresses potential sustainment of TMCU at participating schools. This includes biannual outcome assessments completed by TMCU interventionists, cost-benefit and cost-effectiveness analyses, and elicitation interviews with school leadership.

#### Training outcome assessments

All interventionists complete biannual training outcome assessments (i.e., 6-, 12-, 18-, and 24-months after training). Like preceding assessments, each involves completion of an audio-recorded SP interaction to measure TMCU fidelity, and web-based survey assessment of TMCU knowledge, attitudes, and adoption readiness. A novel component of these follow-up assessments is the inclusion of interventionist-reported satisfaction with technical assistance they receive.

#### Exit interviews

At conclusion of 24-month implementation, two investigative team members (AL, DW) will conduct an elicitation interview with leaders and interventionists at each school. Setting facilitators and barriers to TMCU sustainment will be explored. Like initial interviews with school leadership, exit interviews will be audio-recorded and subject to conventional and directed content analysis [70].

#### Cost analyses

Examination of the value and relative affordability of the two technical assistance conditions will be governed by an investigative team member (KM) with expertise in economic evaluation. This involves use of a standardized survey [74] to estimate annual TMCU costs, incorporating student case-flow data (i.e., average daily student census, number of TMCU sessions delivered) to determine annual cost per student. In comparing costs of the technical assistance methods, data will correspond to aggregate resource use associated with TMCU implementation—including incremental costs associated with each method. Cost analyses will be framed from the school perspective, thus research costs will be excluded. Final products of the analysis will be estimates of total TMCU cost, respective incremental costs of each technical assistance method, annual cost per student, and average cost per TMCU intervention (based on mean number of TMCU sessions completed).

Cost estimates will then be linked with key outcomes to determine cost-benefit and cost-effectiveness. Cost-effectiveness analysis compares differences in the incremental cost of TMCU to differences in its incremental effectiveness in reducing marijuana use to calculate the incremental cost-effectiveness ratio. This ratio describes the additional cost to achieve a unit of desired outcome (i.e., cost per one fewer day of student marijuana use) in TMCU relative to waitlist/control. Cost-benefit analysis broadens the economic impact assessment by comparing intervention costs to the monetary value of reducing negative consequences (i.e., school absences, criminal activity, medical costs, and lost productivity among parents/caregivers. Monetary conversion factors for units of outcome (e.g., cost per criminal offense or cost per day missed from work) translate such outcomes into dollars, which can then be directly compared to intervention costs. The cost-benefit ratio expresses the dollars of benefit generated per dollar invested in the intervention.

### Data management

Trial data consist of audio-recordings and web-based surveys. Audio-recordings include elicitation interviews of school leadership, SP interactions completed by TMCU interventionists, and TMCU sessions with participating students. Web-based surveys are completed by TMCU interventionists, other school-based staff, and students. Upon their collection, audio-recordings will be transported immediately to secure university office space and uploaded as audio-files to a password-protected network and identified by appropriate trial identification number. Original recordings will then be deleted from digital recording devices. Upon their completion, web-based surveys will be stored on encrypted university servers accessible only by trial staff, similarly identified by trial identification number. As outlined in a corresponding Data Safety and Monitoring Plan, linkage between identifying information for trial participants and trial identification numbers will be destroyed at trial conclusion.

Planned reliance on web-based surveys for most of the trial data collection limits the need for data entry procedures. An exception is paper forms corresponding to audio-recording data (i.e., TMCU fidelity ratings), for which numeric data will be entered and later reviewed for verification by separate study staff. Likewise, automated entry of web-based survey data will be reviewed for verification by study staff who will identify and fix anomalies. Record of data anomalies will be maintained in a trial logbook. To facilitate data completeness, a data tree will be established in order that data is analyzed and stored in specific files, backed up in secure locations on a weekly basis.

### Hypothesis testing and analytic approach

Hypotheses focus on comparative utility of two technical assistance methods on implementation outcomes among TMCU interventionists and intervention effectiveness among students. Hypothesized effects are that the (1) longitudinal effects of interventionists receiving ‘gold-standard’ vs. ‘as-needed’ technical assistance will not differ, with the latter method proving more cost-beneficial and cost-effective; and (2) effects of technical assistance method for intervention impact on student marijuana use frequency will not differ, with greater reduction expected of students randomly assigned to TMCU vs. waitlist/control.

Trial analyses will be governed by an investigative team member (KK), experienced as a biostatistician and methodologist in adolescent health research. Given nesting of trial data (i.e., repeated observations of students, nested in interventionist caseloads; interventionists nested in schools), multilevel models will include progressive taxonomies in model-building. Initially, an unconditional model will estimate variance of random effects, and a subsequent unconditional growth model will identify the appropriate shape of and variability in rate of change over time for hypotheses involving repeated measures. Technical assistance condition will then be included in conditional models with predictors, with relevant covariates added to determine robustness of main effects and relative goodness-of-fit for each successive model via likelihood ratio tests and deviance statistics. Power analyses (.80, *p* < .05) inform proposed recruitment of (1) 30 interventionists to detect longitudinal effects of training and technical assistance consistent with those for other lay counselors (Cohen’s *d* = 1.00); and (2) 250 students to detect small intervention effects (Cohen’s *d* = .35) relative to waitlist/control.

#### Hypothesis 1a: comparable utility of technical assistance methods

A non-inferiority paradigm, traditionally used to show a novel treatment is not statistically different than an established one, will test study hypotheses. This paradigm will be applied to interventionists’ longitudinal implementation outcomes, principally TMCU fidelity (i.e., R:Q, %OQ, %CR) in SP interactions but also secondary outcomes of TMCU knowledge, attitudes, adoption readiness, and satisfaction. Initial effects are consistent with training intent, with analyses testing if ‘gold-standard’ and ‘as-needed’ technical assistance produce equivalent longitudinal impacts. If (as expected) the number of schools recruited is small, they will be dummy-coded and treated as fixed effects. Point-estimates and confidence intervals from the model will be included in non-inferiority tests, examining each technical assistance method for (1) pre-post change in implementation outcomes, (2) mean implementation level by TMCU interventionists over time, and (3) differential rate of change in implementation over time. As recommended [75], a ½ standard deviation (SD) difference will serve as the a priori non-inferiority margin. If the lower bound of two-sided 95% confidence interval of between-method differences exceeds this margin, a test of superiority will specify the direction and magnitude of differential impact of the technical assistance methods.

#### Hypothesis 1b: comparable clinical effectiveness of technical assistance methods

For student outcomes, multilevel models extend to three levels for repeated observation of marijuana use frequency and secondary outcomes (i.e., problems, motives, perceptions, self-efficacy to abstain, academic performance, and engagement) nested within interventionists. A primary test parameter is the interaction of time × technical assistance method of the TMCU interventionist as a dummy-coded predictor. As temporal effects may not conform to simple linear growth curves, models will test different specifications of time (i.e., piecewise, polynomial, or other nonlinear approaches). The a priori non-inferiority margin (i.e., ½ SD) will again be applied and, if exceeded, a test of superiority will specify the direction and magnitude of differential impact.

### Trial regulation and current status

In accordance with National Institute on Health policy, a Data Safety and Monitoring Board (DSMB) was assembled to whom the principal investigators (BH, DW) recurrently report trial progress. Content expertise of this three-member, university-based DSMB encompasses school-based health service implementation, brief interventions, and community-participatory addiction research. The trial coordinator (LM) received approval for all procedures from the University of Washington Institutional Review Board to whom protocol modifications and adverse events will be reported, obtained a Certificate of Confidentiality, and registered the trial at ClinicalTrials.gov (NCT03111667). As for trial progress, recruitment of local schools has been initiated with formal enrollment of TMCU interventionists expected in the months to come.

## Discussion

As schools are a common point-of-contact for students in the USA to access behavioral health services [21, 22], there are several reasons this has potential to address marijuana use. First, most adolescents who misuse substances evidence marijuana use disorder [7], a leading impetus for youth treatment admissions [8]. Second, progressive legislation of medical/recreational marijuana use widens its public availability and cultural permissibility [13], while risk perceptions among high school students continually decline [12]. Third, over 95% of teens with a substance use disorder continue to attend school—so school-based services should offer significant reach [76]. Taken together, these issues underscore need for effective, affordable strategies to support transport of efficacious, marijuana-focused health services for use by school-based personnel. The described trial—and its testing of a fidelity alarm bell as a method of purveyor technical assistance—is designed to offer one such effort. Resulting trial findings are likely to hold implications for similar service transportability challenges across a range of health services and the community-based settings for which they are appropriate.

### Innovation and potential impact

The described trial offers innovation at multiple levels. Most broadly, its findings will address a salient gap in implementation science literature through examination of processes for purveyor introduction and support of an empirically supported health service. Though this trial will specifically examine implementation of a brief marijuana-focused intervention in schools, replication of many design features described herein may be appropriate for other trials. At the level of implementation strategies, this trial will test a novel approach whereby technical assistance is provided to school-based staff after initial training. As technical assistance processes remains an understudied topic [77], a clinically useful, affordable means to provide such support may hold considerable impact for therapy purveyors and community settings. Notably, the ‘fidelity alarm bell’ to be tested retains core elements (i.e., purveyor fidelity monitoring, feedback, coaching) recommended for implementing MI/MET [41] and other therapies [39, 40]. A final point concerns potential future interplay with ongoing validation work on automated MI/MET fidelity assessment methods that code session recordings via computer and transform fidelity indices as visual feedback [78]. If feasible, future work may pair a fidelity alarm bell with these promising natural language processing approaches to streamline fidelity-monitoring processes [79–81] and thereby boost the efficiency and scale with which they occur. Diminished purveyor burden in this area may open possibilities to enhance feedback/coaching processes [82], or simply reduce purveyor reliance in favor of localized, self-sustaining quality assurance methods [48].

### Limitations

Design caveats bear mention. Potential for selection bias exists for participating schools, interventionists, and students. Recruitment of schools that collaborated on prior TMCU trials may undermine generalizability of results, though both the multisite design and broad inclusion criteria for settings and interventionists mitigate this concern. With respect to TMCU effectiveness, student inclusion/exclusion criteria are consistent with those of prior efficacy trials and the intent of this targeted intervention [27]. The trial responds to local demands in Washington state, where legalization of recreational marijuana use among adults has amplified public concern and demand for preventative services. Persisting variation in legislative marijuana policy, both domestically and abroad [83], suggests this local context be considered in eventual interpretation of trial findings. Measurement choices present additional caveats. Trial aims necessitate audio-recording of TMCU sessions, which for some may introduce social anxiety and impression management. These concerns appear not to extend to planned SP interactions, given strong evidence for valid, reliable MI fidelity estimates via this methodology [84]. Reliance on student-report instruments to examine TMCU effectiveness is another caveat, particularly for marijuana use—though use of invasive biological testing with minors would introduce greater scientific and pragmatic concerns. As in any trial, potential influence of unassessed third variables is a possible limitation. Further, alternative trial designs (i.e., cluster-randomization, stepped-wedge) may offer some potential benefits in efficiency, albeit not without introducing other unwanted caveats.

## Conclusions

This trial offers potential advancement for the implementation science field, specifically in its thorough examination of a data-driven technical assistance method to support long-term implementation of a marijuana-focused MET intervention in schools. If shown to be clinically useful and affordable, the concept of a fidelity drift alarm could be readily translated—alone or in future conjunction with emerging methods to automate MI/MET fidelity-monitoring—for use with other empirically supported therapies and in other settings. The described trial adds to other recent efforts [85] in furthering momentum for the adoption and effective implementation of efficacious therapies for youth populations through a focus on methods of purveyor technical assistance to assure quality in therapy delivery.

## Abbreviations

%CR: Percentage of reflections deemed to be ‘complex’
%OQ: Percentage of questions deemed to be ‘open’
DSMB: Data Safety Monitoring Board
EPIS: Exploration, Preparation, Implementation, Sustainment
MET: Motivational enhancement therapy
MI: Motivational interviewing
MINT: Motivational Interviewing Network of Trainers
R:Q: Ratio of reflections to questions
SD: Standard deviation
SP: Standardized patient
TMCU: Teen Marijuana Check-Up
